# Soleus H-Reflex Change in Poststroke Spasticity: Modulation due to Body Position

**DOI:** 10.1155/2021/9955153

**Published:** 2021-12-07

**Authors:** Wenting Qin, Anjing Zhang, Mingzhen Yang, Chan Chen, Lijun Zhen, Hong Yang, Lingjing Jin, Fang Li

**Affiliations:** ^1^Department of Rehabilitation Medicine, Huashan Hospital, Fudan University, Shanghai, China; ^2^Department of Neurology, Shanghai Tongji Hospital, Tongji University School of Medicine, Shanghai, China; ^3^Department of Neurorehabilitation Medicine, Kongjiang Branch, the First Rehabilitation Hospital of Shanghai, Yangpu District, Shanghai, China; ^4^Department of Rehabilitation Medicine, Zhongshan Hospital, Fudan University, Shanghai, China; ^5^Department of Rehabilitation Medicine, Renhe Hospital, Baoshan District, Shanghai, China; ^6^National Center for Neurological Disorders NCND, Shanghai, China

## Abstract

**Purpose:**

This study is aimed at exploring how soleus H-reflex change in poststroke patients with spasticity influenced by body position.

**Materials and Methods:**

Twenty-four stroke patients with spastic hemiplegia and twelve age-matched healthy controls were investigated. Maximal Hoffmann-reflex (Hmax) and motor potential (Mmax) were elicited at the popliteal fossa in both prone and standing positions, respectively, and the Hmax/Mmax ratio at each body position was determined. Compare changes in reflex behavior in both spastic and contralateral muscles of stroke survivors in prone and standing positions, and match healthy subjects in the same position.

**Results:**

In healthy subjects, Hmax and Hmax/Mmax ratios were significantly decreased in the standing position compared to the prone position (Hmax: *p* = 0.000, Hmax/Mmax: *p* = 0.016). However, Hmax/Mmax ratios were increased in standing position on both sides in poststroke patients with spasticity (unaffected side: *p* = 0.006, affected side: *p* = 0.095). The Hmax and Hmax/Mmax ratios were significantly more increased on the affected side than unaffected side irrespective of the position.

**Conclusions:**

The motor neuron excitability of both sides was not suppressed but instead upregulated in the standing position in subjects with spasticity, which may suggest that there was abnormal regulation of the Ia pathway on both sides.

## 1. Introduction

Spasticity is defined as velocity-dependent increase in the tonic stretch reflexes (muscle tone) [1], which is one of the positive signs of upper motor neuron syndrome. It can cause continuous contraction of the affected muscles and difficulty in muscle coordination. Spasticity can cause pain, limited active and passive activities, and difficulty in nursing, which will seriously reduce the quality of life of the patient. Notably, as reported by Zorowitz et al. [2], the estimated range of spasticity in stroke survivors ranges from 20% to 40%.

In clinical practice, spasticity is usually measured at rest, based on the combination of physical signs, including increased muscle tone and tendon hyperreflexia. A previous study conducted by our research group showed that from the supine position to standing or sitting position, Modified Tardieu Scale (MTS) and Triple Spasticity Scale (TSS) scores significantly increased in the evaluated muscles of the hemiplegic upper limb in all poststroke patients [3]. Wang et al. observed that the contraction rates of elbow flexors in the affected side increase with the difficulty in different standing postures [4]. De Azevedo et al. found that in SCI patients a change in body position from sitting to supine increased the spastic state in quadriceps femoris during the pendulum test [5]. All above studies indicated that posture plays an important role in the spasticity regulation.

Burke et al. believed that the loss of homosynaptic inhibition between the Ia afferent pathway and motor neurons changes in reflex circuits affecting motoneuron excitability (e.g., Renshaw inhibition, Ib inhibition, and reciprocal Ia inhibition), and the intrinsic property changes of motor neurons and muscles make up the leading causes of increased muscle tone and tendon hyperexcitability in spastic patients [6]. The effect of posture on spasticity suggested that the spinal reflex mechanism in spasticity during motion might not be identical with rest. Importantly, only a few spinal mechanisms can explain the excessive muscle activity at rest, whereas almost all spinal cord circuits participate in defective spinal cord control in the state of motion, however, resulting in dyskinesia [6, 7].

Several studies showed that spastic patients exhibited a larger H-reflex amplitude or Hmax/Mmax ratio than the control group or their unaffected side in the prone position [8], suggesting that spasticity at rest results from the increased excitability of motor neurons in spinal circuits. Cattagni et al. and Kim et al. found that the Hmax/Mmax ratio can be affected by the muscle activity or body position in healthy individuals [9, 10]. This phenomenon may be associated with reciprocal inhibition or other suppression mechanisms in the spinal cord level. A particular study by Katz and Pierrot-Deseilligny investigated the recurrent inhibition by conditioning H-reflex changes in spastic patients during various voluntary and postural contractions. The study concluded that paralysis of the supraspinal control of Renshaw cells might partly account for muscular debilitation and dexterity loss of voluntary movement in spastic patients [11]. Though there are only a few studies on H-reflex in spastic patients at a standing position or movement, the exact pathology of spasticity is still unknown.

Accordingly, the objectives of this study were as follows: (1) if varying posture could influence H-reflex excitability on both sides in stroke patients with lower limb spasticity and (2) to compare changes in reflex behavior in both spastic and contralateral muscles of stroke survivors and match healthy subjects in a standing position. We hypothesized that posture transformation would affect the H-reflex excitability of spastic patients on both sides. Our theory would thus help us understand that the role modulation of afferent Ia spinal cord pathway plays in a standing position. Also, if the hypothesis proves right, it would enable us to explore further the pathophysiological mechanism(s) of spasticity in a standing position. We also hypothesized that unstable posture might acutely increase spinal cord excitability.

## 2. Materials and Methods

### 2.1. Participants

We recruited a total of twenty-four hemiparetic spastic stroke survivors in the rehabilitation inpatient department in Huashan Hosipital and twelve age-matched healthy subjects (patient's caregiver) serving as controls. Inclusion criteria for the stroke subjects were (1) hyperactive ankle jerk with clonus and extensor plantar response (Babinski response), (2) present range of motion (ROM) difference between two speed and the quality of ankle joint plantar flexor muscle reaction elicited on the fast passive stretch ≥ 2 according to the Modified Tardieu Scale (MTS), (3) Brunnstrom recovery stage 3 or 4 in the lower limb, (4) the ability to stand independently, and (5) the presence of soleus H-reflex. Exclusion criteria were (1) with history of Parkinson's disease or other neurological diseases; (2) taking any medication that may affect motor control or nerve function, such as Baclofen; (3) medically unstable or concurrent uncontrolled systemic illnesses; (4) skin ulceration, irritation, and inflammation in bilateral calves which influence placing electrodes; and (5) severe disturbance of emotion, visual, and cognitive impairment. All participants gave informed consent via the study protocol approved by the ethics committee of Huashan Affiliated Hospital, Fudan University (ChiCTR-TRC-1800018427).

### 2.2. Procedures

Before the beginning of this study, demographic information such as gender, age, height, and stroke-related information such as the course of disease, hemiplegic side, and stroke types was also collected.

### 2.3. Electrophysiologic Evaluation

The H-reflex and M-wave recruitment curves in the soleus muscles of each patient were recorded three times by electrodiagnostic equipment (Shanghai Nuocheng Electrical Co., Ltd.; Shanghai, China) at the fixed time (8 : 00 am-9 : 00 am) before the rehabilitation on the same day. The recruitment curves were carried out in two different body postures: prone position and standing position. In the prone position, the subjects comfortably laid on the treatment bed face down, while a triangular sponge pad was placed under their ankle joint to relax the calf triceps. In standing position, however, participants were asked to stand still, with their head and the upper body in a neutral position, eyes straight ahead, feet apart with the toes facing forward, and the upper limbs hanging on both sides of the torso. The center of gravity of the body is located between the feet and cannot be deflected. The evaluation was as follows: the unaffected side in a prone position, the affected side in a prone position, the unaffected side in a standing position, and the affected side in a standing position. After the tests in a prone position, each participant was given a five minutes break for which the subject's position was then changed. The self-adhesive surface electrodes (2.0 cm Ag-AgCl square electrodes) were attached to the belly of Soleus (active electrode) and the Achilles tendon (reference electrode). A ground electrode (2.0 cm Ag-AgCl square electrode) was attached to the skin between the active electrode and the reference electrode. The tibial nerve was stimulated at the popliteal fossa using a handheld bipolar stimulator (1 ms rectangular pulse), with the cathode was pointing towards the proximal end. Where the stimulator electrodes were to be placed on the skin was marked with an indelible pen to ensure that the same recording site was used in the successive session. The optimal stimulation site of the posterior tibial nerve was identified using the stimulating electrodes.

The H-reflex (Hmax) was determined using many stimuli as required at 0.5 mA, precisely around the intensity eliciting the largest amplitude of the H-reflex. The maximum amplitude of the M-wave (Mmax) was determined as the size of the response to a stimulus of supramaximal intensity. Amplitude was measured from baseline to the largest negative peak in each series while determining the Hmax/Mmax ratio. Recording parameters settings are as follows: the sensitivity is 2 mV, the scanning speed is 5 ms/cm, and the stimulation intensity duration is 1 ms. The EMG signal was amplified (1 mV/D) and band-pass filtered (2–10,000 Hz).

### 2.4. Data Analysis

The H-reflex (Hmax, Mmax, Hmax/Mmax ratio) from each subject were presented as mean ± SD. One-way ANOVA was employed to assess the differences in H-reflex data among the affected side, unaffected side in spasticity patients, and the average value of both sides in healthy subjects. The H-reflex results of the same side in stroke patients with spasticity in different positions were tested using a paired-sample *t*-test (assuming a normal distribution) and using Kruskal-Wallis (nonparametric test) to test if it does not meet the normal distribution.

## 3. Results

The demographic and clinical characteristics of all participants are summarized in Table 1. In the healthy subjects, the mean age was 55.17 ± 4.67 years, ranging from 41 to 66 years of age. The mean age of stroke patients was 54.54 ± 12.36 years, ranging from 49 to 71 years. The mean time since stroke outset was 6.17 ± 6.72 months, ranging from 1.5 to 30 months. There were no significant differences in age, gender, or height between the two groups.

### 3.1. Comparison of H-Reflex Results between the Affected Side and the Unaffected Side in the Same Position

**Figure 1:**
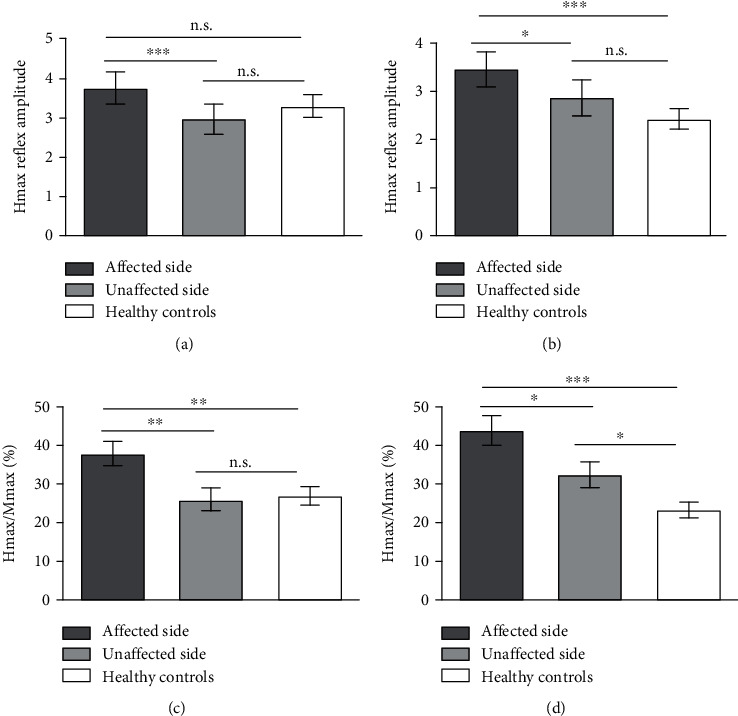
The post hoc multiple comparisons for the H-reflex data of the affected side, the unaffected side, and the healthy control group: (a, c) in the prone position; (b, d) in the standing position. Asterisks indicate significant differences. ns: not significant. ^∗^*p* < 0.05; ^∗∗^*p* < 0.01; ^∗∗∗^*p* < 0.001.

In the prone position, post hoc multiple comparisons were used for the H-reflex data of the affected side, the unaffected side, and the healthy group. The mean ± SD of the Hmax for the affected side, the unaffected side, and healthy control groups were 3.75 ± 1.95 mV, 2.96 ± 1.92 mV, and 3.28 ± 1.44 mV, respectively. Significant differences existed between the affected side and the unaffected side (*p* = 0.0002), but the differences between the affected side and the healthy subjects as well as the unaffected side and the healthy subjects were not statistically significant (Figure 1). The Hmax/Mmax ratio of the affected side (37.95 ± 15.16%) was significantly higher than the unaffected side and the healthy subjects (25.91 ± 13.61% and *p* = 0.003 and 26.88 ± 11.88% and *p* = 0.006, respectively). Nevertheless, no statistical difference in the Hmax/Mmax ratio was found between the unaffected side and the healthy subjects.

In the standing position, the mean ± SD of the Hmax for the affected side, the unaffected side, and healthy control groups were 3.46 ± 1.69 mV, 2.88 ± 1.84 mV, and 2.41 ± 0.97 mV, respectively. Significant differences existed between the affected side and the healthy subjects (*p* < 0.0001) and the affected side and the unaffected side (*p* = 0.038). Nevertheless, the differences between the unaffected side and the healthy subjects were not statistically significant. The mean ± SD of the Hmax/Mmax ratio for the affected side, the unaffected side, and healthy control groups were 43.58 ± 19.05%, 32.32 ± 16.23%, and 22.95 ± 9.58%, respectively. The statistical differences for the Hmax/Mmax ratio were found between any two groups in the standing position.

### 3.2. Effects of Different Postures on the Results of H-Reflex in Healthy Subjects and Stroke Patients with Spasticity

**Figure 2:**
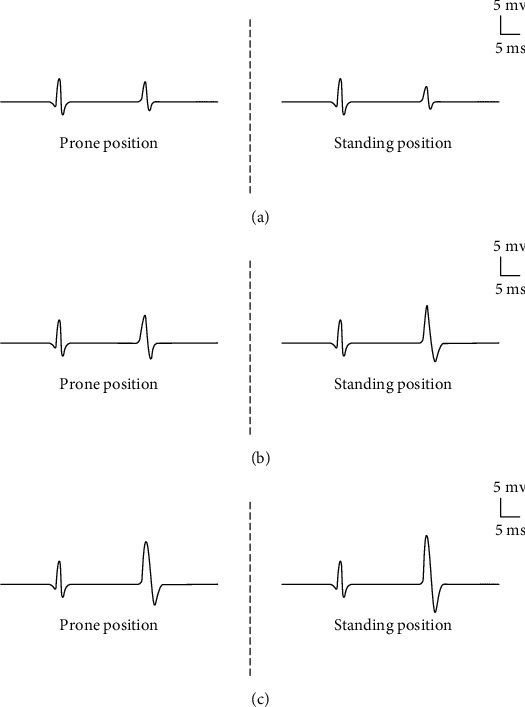
The Hmax in different positions: (a) healthy subject; (b) the unaffected side in a stroke patient; (c) the affected side in the same stroke patient (left: prone position; right: standing position). The first wave means the M-wave, and the second wave represents the Hmax. Note the same amplitude changing trend of the Hmax in the both conditions for the patient.

In healthy subjects, Hmax significantly decreased in standing position compared to the prone position (Figure 2(a)). In stroke patients with spastic hemiplegia, the Hmax, however, did not decrease but somewhat increased in the standing position on both sides in several patients (14/24 for the Hmax) (Figures 2(b) and 2(c)).

**Figure 3:**
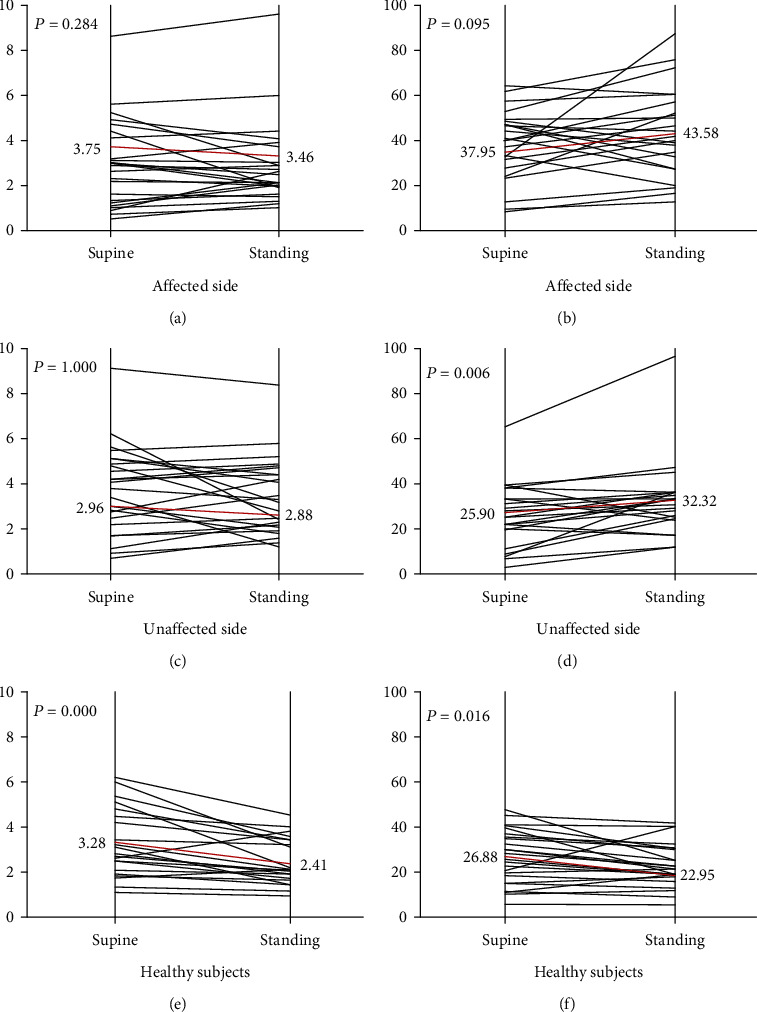
The individual results of H-reflex on the affected side (a, b), the unaffected side (c, d) of stroke patients, and in healthy subjects (e, f) from the prone position (left panels) to the standing position (right panels). (a, c, e) Hmax. (b, d, f) Hmax/Mmax ratio. The individual values (thin line) and mean (thick red line, values indicated beside the vertical line) obtained on the affected and unaffected sides from 24 stroke patients and 12 healthy subjects.

In healthy controls (Figure 3), the mean values of the Hmax (e) and the Hmax/Mmax ratio (f) were significantly higher in the prone position than in the standing position ((e): *p* = 0.000, (f): *p* = 0.016). Individual results on the affected side of most stroke patients showed that the data of H-reflex (a, b) in prone position were lower than in the standing position (14/24 for the Hmax and 20/24 for the Hmax/Mmax ratio), however, which showed no statistical difference in the mean value of the Hmax ((a): *p* = 0.284) and the Hmax/Mmax ratio ((b): *p* = 0.095) from the prone position to the standing position in patients. On the unaffected side, the mean value of the Hmax/Mmax ratio was significantly larger in the standing position than in the prone position ((d): *p* = 0.006).

## 4. Discussion

The primary observation in the present study is that a transformed position can significantly alter spinal cord excitability in stroke patients with spasticity. In neurologically intact individuals, a striking decline could be found in Hmax and Hmax/Mmax ratios from the prone to the standing position. Notably, in the participants with lower limb spasticity, the H-reflex was not suppressed on both sides during postural adjustment. Regardless of the prone and the standing position, the affected sides exhibited distinctly increasing in terms of spinal excitability, and the Hmax/Mmax ratio in the unaffected side was significantly higher in the standing position. It suggests that the neural mechanisms responsible for posture adjustment may be different between stroke patients with spasticity and the healthy subjects.

The pathophysiological mechanism of spasticity after stroke is complicated and still being explored. It is currently believed that the cause of stretch reflex hyperexcitability in stroke patients with spasticity is primarily due to abnormal remodeling of the descending conduction pathway above the spinal cord level and error processing within the spinal cord level [12]. All these changes could affect the excitability of alpha motor neurons [13]. The excitability of alpha motor neurons could represent the severity of spasticity to some extent. Thus, H-reflex, reflecting the excitability of alpha motor neurons, has been used to investigate the role of peripheral sensory afferent or supraspinal descending conduction pathway in various aspects of human movements [14].

In the present study (Figure 2), when a healthy person changed from a prone position to a standing position, the Hmax significantly decreased. The results showed that the excitability of the related alpha motor neuron and the Ia afferent pathway were modulated during standing. The study results by Cattagni et.al showed that the Hmax/Mmax ratio in an active sitting was lower than in a passive sitting and lowest in an upright standing position [9]. In another study, H-reflex amplitudes of fibularis longus (FL) and soleus in healthy subjects were significantly lower in the uni-pedal stance than the prone and bipedal positions [10]. We believed that the excitability decrease of H-reflex in healthy people might be associated with reciprocal inhibition in the spinal cord level, which may also involve others spinal cord suppression mechanism [15].

Compared to the healthy subjects in standing positions, the H-reflex amplitude of the affected side distinctly increased in the stroke patients with spasticity (Figures 2 and 3). The present study indicated that the spastic hemiplegic patients had an overall upregulation of the anterior horn cells excitability both in the prone and the standing positions, which is also in line with previously reported findings [16]. Fleuren et al. stated that when the patients changed their body position from a prone position to that of a standing position, their triceps surae muscle was lengthened. The increased muscle length could augment the stretch reflex activity [17]. The tonic activity from the proprioceptors may presynaptically interfere with the effectiveness of the spindle primary afferent synapses on the soleus motor neurons [14]. Importantly, the Hmax/Mmax ratio appears to be exclusively modulated by body position (Figure 3). This body position transition might deteriorate spasticity, which shows that the abnormal reciprocal inhibition of Ia fiber exists in spastic patients.

In the present study, we found out that the H-reflex excitability was not inhibited on both sides, and the anterior horn cell's excitability of the unaffected side was even enhanced in the standing position in spastic patients. It also indicates that the abnormal Ia reciprocal inhibition in these patients can occur on both sides. The results of the present study are in line with the study by Phadke et al. [18]. On the contrary, Kawashima et al. reported that the soleus H-reflex from sitting to standing was inhibited in patients with complete SCI [19]. In addition to different testing conditions, another critical issue with their study was that it failed to take spasticity into account.

Besides the intraspinal processing, the reticulospinal tract (RST) might be taken into consideration. The RST is best known for playing an essential role in maintaining joint position, posture against gravity, and locomotion [20]. Reticulospinal pathways usually have bilateral projections for head, neck, trunk, and proximal limb movements [21]. The hyperexcitability of RST could be found in the spastic stage but not in recovered nonspastic stages [22, 23]. The increased stretch reflex thresholds of the biceps brachii muscle on both contralateral and affected sides of stroke survivors suggested that RST activation in the spastic stage still have a bilateral descending influence [24]. The increased Hmax/Mmax ratio on bilateral sides in stroke patients with spasticity was observed at rest and during standing in the present study. Motor overflow from the affected limb to contralateral side extended the findings of all abovementioned studies and may further support reticulospinal hyperexcitability at least partially responsible for increased stretch reflex excitability. However, for studies on other pathways within the spinal cord level or supraspinal level during movement, extensive electrophysiological methods are needed.

In healthy people, there may be a positive correlation between the depression or downward modulation of the soleus H-reflex and the degree of the postural instability in which the subjects are placed [25]. The depression modulation of SOL H-reflex under such condition is predominately a result of the presynaptic inhibitory mechanisms for avoiding oversaturation of the spinal motoneurons to obtain more descending commands for postural correction [26]. In spastic patients, the ascending sensory and descending supraspinal tracts are interrupted in different degrees. The decreased suppression of the stretch reflex and the delayed or reduced spinal reflex processing may contribute to impaired balance control [27, 28]. Therefore, the worse the balance function of the spastic patients, the less the depression modulation of the H-reflex in the standing position. We inferred that the impaired Ia afferent pathway might be the cause of the postural control defects in spastic patients. In the standing position, the abnormal Ia spinal cord afferent pathway participates in the defective spinal cord control, which not only aggravates the severity of the spasticity but might also exacerbate motion control.

This study has some limitations. First, we found a clinical phenomenon which might be explained by RST, but we did not verify this hypothesis. Therefore, our further study will focus on exploring the underlying mechanisms in stretch reflex hyperexcitability involving the role of RST using indirect noninvasive measure such as acoustic startle reflex (ASR) to make a further investigation about the spinal or supraspinal mechanism of H-reflex changes in standing position. Secondly, the current study was based on a small sample of participants. Despite it, we still observed the H-reflex difference between the two postures in the same patient.

## 5. Conclusions

In standing position, the H-reflex excitability of the tibial nerve is partially upregulated in stroke patients with spasticity on both sides. The findings also suggested that the impaired regulation of Ia afferent pathway may be a potential source of postural control defects in poststroke spasticity.

## Figures and Tables

**Table 1:** Demographic and clinical characteristics of the subjects.

	Stroke group (*n* = 24)	Healthy group (*n* = 12)	*p* value
Age (years)	54.54 ± 12.36	55.17 ± 4.67	0.868
Gender (M/F)	16/8	5/7	0.635
Height (cm)	168.21 ± 6.30	165.42 ± 6.52	0.227
Affected side (R/L)	10/14		
Time since stroke (months)	6.17 ± 6.72		
(CI/CH)	11/13		

M: male; F: female; CI: cerebral ischemia; CH: cerebral hemorrhage; L: left; R: right.

## Data Availability

The data that support the findings of this study are available from the corresponding author upon reasonable request.
